# Modern Views of Malaria. II

**Published:** 1894-05-05

**Authors:** 


### Modern Views of Malaria.—II.

In our last article we described the parasite which, living in the blood, causes ague, and we showed that it is an amœba-like body undergoing a developmental cycle.

The incubation period is naturally a matter of very great interest. It is probably very long, as the following quotation from Laveran will show: "A body of French troops traversed localities known to be unhealthy, and no case of paludism occurred. However, only a few days after arrival at Constantine, where the men were located in very healthy barracks, symptoms of paludism appeared in a large number of men; certain patients were seized a month or more after their arrival, whilst others were attacked a month or more after crossing the marshy districts." Then again, most doctors will be able to recall from their practice cases in which patients have been to malarious districts without suffering at all, but who have, when they returned to England, been found on some slight provocation to develop a typical attack of ague. We must therefore conclude that the latest period of the malarial poison is very variable, but that the extreme limit is at present undetermined. It is clear, therefore, that the hæmatozoon of ague may remain undeveloped in the blood of patients for an almost indefinite time.

The next question to be considered is how do these parasites, when introduced into the blood, produce the phenomena of ague. The most constant, and the most important symptom of malarial infection is anæmia, and now the cause of this is easy to see, for it clearly depends upon the fact that the parasites in the blood live at the expense of the red corpuscles in it; in fact, no anæmia excepting that of hæmorrhage can be better explained than that of malaria. This destruction of the red blood discs also shows us why it is so frequent to meet with masses of pigment in the smaller blood vessels of those who have died from malaria. Sometimes all that the parasite does is to produce anæmia, and none of the other symptoms of ague are present, for it is quite common to meet with people who, as a result of living in an aguish district, are very anæmic, but they have none of the ordinary symptoms of ague. The regular periodicity of the attacks of ague is difficult of explanation, and in connection with it we must remember that in the first place it is not constant, and in the next place it occurs in many other conditions, thus the filaria sanguinis hominis produces regular periodical attacks; periodicity is noticed in the hectic of phthisis, in certain liver diseases, and in neuralgias. It is probably in some way connected with the stages of development of the parasite.

As the spleen is so constantly enlarged in ague it is only natural that much attention has been attracted to it, and Councilman and other observers have by means of a fine syringe drawn blood from it between and during the attacks of ague, and have always found that the parasites of ague are much more abundant in this blood than in that drawn from other parts of the body and that the flagellate forms are particularly numerous. The spleen is thus a regular hotbed for the malarial parasites, and thus we see the explanation of the well-known fact that the spleen of those who succumb to ague is always found to be large and reduced to a mere pulp. On examining it histologically abundance of pigment is found and many of the crescentic bodies we have previously described. The abundance of parasites in the general circulation varies at different times, and is greatest during an attack; thus ague seems to be like filarianous disease and relapsing fever, for in the former of these the filaria and in the latter the spirillum retires between the attacks into the deeper vessels, and only appears in the superficial ones during an attack, but the analogy is not quite complete, for in ague some parasites may be found in the blood between the attacks.

The rise of temperature is almost certainly due to some toxine produced by the parasite. What the toxine is we do not know, but it is stated that the urine of patients suffering from a paroxysm of ague is much more poisonous than urine usually is. Now let us see if we can learn anything about the mode of recovery from ague. Many persons will undoubtedly recover even if they never have quinine. Metschnikoff has especially studied relapsing fever, and has shown that a veritable battle takes place between the spirillum and the phagocytes, it lasts as long as the former are free in the blood, and ends when they have become a prey to the phagocytes. He has only studied a few cases of malaria, but as might have been expected the same explanation holds here, for he says: "In studying the internal organs of two fatal cases of malarial fever I have been able to convince myself in a very marked manner of the action of the phagocytes in this disease. It is principally those of the liver and spleen which absorb quantities of malarial parasites often in surprisingly large numbers." Thus we see that natural cure in malaria is essentially a process of phagocytosis.

With regard to treatment, the victory of the phagocytes in the battle will be greatly aided by putting the patient in the best possible general conditions of health, such as giving him plenty of fresh air and plenty of fresh food, and, above all, change of climate.

But the great drug, as is well known, is quinine. Cinchona was used and much appreciated in England under the name of Peruvian bark as far back as 1660, and Pelletier subsequently isolated quinine from it. Much laborious work has been expended in studying the action of quinine on the healthy human organism, but now that we know that ague is due to a definite protozoan parasite, we can understand why such researches led to such slight results. The action of quinine on the hæmatozoon may be studied directly by mixing a drop of solution of sulphate or hydrochlorate of quinine with a drop of blood from an ague patient, when it will be noticed that the flagella are no longer observed, and the hæmatozoa assume their dead form. Another clear proof of the action of quinine is the fact that the protozoa disappear from the blood of patients taking quinine. Probably the whole explanation of the success which attends the use of quinine lies in this power that it has, but it has been suggested that to some extent it acts by aiding directly the process of phagocytosis. For all ordinary purposes the quinine should be given by the mouth in the form of the hydrochlorate, for not only is this more soluble than the sulphate, but it contains 81 per cent. of quinine, and the sulphate contains only 59. The salicylate of quinine has been much recommended, but it is very insoluble and contains only 70 per cent. of quinine. The hydrochlorate of quinine, especially the basic hydrochlorate, may be given subcutaneously, and for this purpose the kinate may also be employed. It is very rare, indeed, for the symptoms of quinine poisoning to be sufficiently severe to render it wise to discontinue it in cases of ague, still the possibility of this being necessary should always be borne in mind. The question has often been discussed as to the best time to give quinine; the majority of authors are agreed that it is best given during the period of apyrexia, for then it excites vomiting less often and the absorption of it is more complete. Cinchinine is far less efficacious than quinine, and antipyrin and eucalyptus, both recommended, do not act on the parasite of ague, although the planting of eucalyptus trees in ague districts does much to remove the intensity of the disease.

